# Clinico-hysteroscopic analysis of severe intrauterine adhesions among Nigerian infertile women

**DOI:** 10.11604/pamj.2017.28.226.13838

**Published:** 2017-11-14

**Authors:** Joseph Odirichukwu Ugboaja, Charlotte Blanche Oguejiofor, Anthony Osita Igwegbe

**Affiliations:** 1 Department of Obstetrics & Gynaecology, Nnamdi Azikiwe University Teaching Hospital Nnewi, Nigeria

**Keywords:** Severe, intrauterine adhesions, hysteroscopy, hypomenorrhea, amenorrhea, Nigeria

## Abstract

**Introduction:**

Severe intrauterine adhesions are difficult to manage and are associated with poor reproductive outcomes following treatment. The objective was to study the clinical presentation and hysteroscopic findings of severe intrauterine adhesions seen at hysteroscopy in two fertility/gynaecological endoscopy units in Nigeria.

**Methods:**

A prospective study of 19 out of 76 women managed for intrauterine adhesions in our units. Data were analyzed with STATA software, version 12.0 SE (Stata Corporation, TX, USA).

**Results:**

Severe intrauterine adhesion accounted for 19 (25.0%) of 76 cases of intrauterine adhesions managed during the period. This constituted 11.9% of 160 infertile women who had diagnostic hysteroscopies in our units over the study period. The mean duration of symptom was 4.2 years +/-3.2. Amenorrhea in association with infertility (68.4%) was the main presenting complaint. Secondary dysmenorrhea and cyclical abdominal pain were found in 10.8% and 31.6% of the women respectively. The main aetiological events were complicated caesarean section (42.1%) and abdominal myomectomy (26.3%). The adhesions were mainly dense (52.6%) and multiple (94.7%) with complete involvement of the uterine cavity in all the cases. Obliterative lesions were seen in 63.2% of the women.

**Conclusion:**

The main clinical presentation of severe IUA was amenorrhea and infertility while the major risk factors were complicated caesarean section and myomectomy. The adhesions were mainly multiple, dense, obliterative and complete.

## Introduction

Intrauterine adhesion (IUA) is a significant cause of infertility and reproductive failure in Nigeria and the prevalence ranges from 2.7% to 8.1% of all gynaecological cases seen [1-4]. Among infertile women being worked up for In-vitro fertilization and embryo transfer, prevalence rates at hysteroscopy range from 49.1% to 64.2% [5, 6] Intrauterine adhesions usually result from post-traumatic or post infectious injury to the basalis layer of the endometrium leading to healing by fibrosis with the resultant obliteration of the uterine cavity. The common risk factors in Nigeria include dilatation and curettage for pregnancy related indications, complicated caesarean section, myomectomy and pelvic inflammatory diseases [3-8]. The diagnosis of IUA can be made by Hysterosalpingogram (HSG), Ultrasonography [9, 10] or hysteroscopy. But hysteroscopy is the gold standard for the diagnosis. There are many classification systems for IUAs but the grading system of the American Society for Reproductive Medicine (ASRM) [11] has been the most popular. It grades IUAs into mild, moderate and severe depending on the assigned scores that are based on the nature of the adhesions, the extent of cavity involvement and the menstrual pattern. Severe IUAs are characterized by extensive involvement of uterine cavity. They are defined by an ASRM score of 9-12 [11]. The key goals of management of severe intrauterine adhesions are restoration of normal uterine cavity, prevention recurrence of adhesions, normalization of menstrual flow and improvement of reproductive outcome. Hysteroscopic Adhesiolysis is the procedure of choice in excision of the adhesion bands. Although studies have shown good success rate in restoration of normal endometrial cavity [12-15], reproductive outcomes have been poor [16-18]. The main challenges in hysteroscopic adhesiolysis in cases of severe IUAs include difficulty in the excision of the dense adhesion bands, as well as prevention of recurrence. Ultrasound and laparoscopy guided procedures have been shown to facilitate complete surgical excision of adhesion bands as well reduction in complication rates [19-21]. Many methods have been devised to facilitate hysteroscopic adhesiolysis in cases of severe intrauterine adhesions but myometrial scoring in which 6 to 8 4-mm deep incisions are created in the myometrium using a resectoscope fitted with a Collins knife electrode from the fundus to the cervix to the isthmus to create a cavity in women with total obliteration of the cavity has also been particularly found to be effective in restoring reproductive function and associated with minimum complications [22]. Strategies that have been shown to reduce the rate of recurrence and improve reproductive outcomes in cases of severe IUAs include the use of paediatric foleys catheter, amnion graft applied over the foleys catheter balloon [23, 24]; use of oxidized, regenerated cellulose adhesion barrier(intercede) alongside an IUCD [25], as well as the use of estrogen for endometrial regeneration [26]. In Nigeria, there is paucity of data on the hysteroscopic management of severe intrauterine adhesions [1-5, 7]. This study was therefore, undertaken to evaluate the clinical presentation and hysteroscopic characteristics of severe intrauterine adhesions among a cross section of Nigerian women.

**Objectives**: The main objective was to study the clinical presentation and hysteroscopic characteristics of severe intrauterine adhesions among a cross section of Nigerian women. The specific objectives include to study the socio demographic characteristics of the women with severe intrauterine adhesions, the clinical presentations of the women with severe IUA seen among the women, the risk factors/aetiological events for severe IUA seen among the women as well as the hysteroscopic characteristics of severe IUA seen among the women.

## Methods

**Study setting**: The fertility and gynaecological endoscopy units of Nnamdi Azikiwe University Teaching Hospital Nnewi Anambra State, Nigeria and Holy Rosary specialist hospital, Onitsha Nigeria.

**Study design**: A cross sectional descriptive survey of women who were managed for severe intrauterine adhesions in our units over an 18-month's period.( 1st November, 2015-30^th^ April, 2017).

**Study population**: All the women who were suspected of having severe intrauterine adhesions on clinical and radiological evaluations were recruited for the study, after adequate counseling. Those with mild to moderate IUAs and those who withheld consent were excluded from the study.

At presentation, a proforma was used to collect data on all the women who were either diagnosed with or suspected of having severe intrauterine adhesions from clinical evaluation at the clinic. Severe intrauterine adhesions were defined by a score of 9-13 using the ASRM criteria [11]. The information obtained included the biosocial data, the presenting complaint, menstrual pattern, reproductive performance and the aetiological/initiating clinical event. Following clinical evaluation including transvaginal scan and hysterosalpingogram, the patients were scheduled for diagnostic hysteroscopy in the immediate post menstrual phase or at any convenient time for the women who were amenorrheic women. The procedures were done under general anaesthesia and Misoprostol (100ug) was inserted into the posterior fornix a night before the procedure in the nulliparous women to aid cervical os dilatation. During the procedure, a 5.5mm diagnostic hysteroscopy sheath was used to define and map the adhesions following a systematic survey of the cervical canal, cervical os and the uterine cavity including the uterine fundus whenever feasible. This was usually followed by hysteroscopic adhesiolysis with 6.5mm operative sheath using the micro scissor. In cases where this was not possible, a further cervical dilatation was done to accommodate the 9mm monopolar resectoscope and the tough adhesion bands were then resected with the 0 degree loop or colles knife using a monopolar cutting current set @ 60 watts with glycine or 5% d/w as the distension media. As much as possible, all the adhesion bands are resected using the appearance of the fleshy muscular myometrium, presence of bleeding and the internal os as guides to the extent of resection. In very difficult cases with complete obliteration of the cavity, the procedure was carried under ultrasound or laparoscopy guidance. Postoperatively, a paediatric foleys catheter is introduced into the uterine cavity and distended with 3-5mls of sterile water depending on the size of the cavity. Patients were also commenced on combined estradiol valerate tablets for 21 days and medroxy progesterone for the last 10 days. They were given antibiotics and discharged home the same day. The catheter was usually removed after 10 days. At the end of the procedure, the proforma was completed with the findings from diagnostic hysteroscopy and operative details.

**Data analysis**: Data were analyzed with STATA software, version 12.0 SE (Stata Corporation, TX, USA). A simple descriptive analysis was done in which the mean and mode were calculated for the continuous variables while the frequencies of the composite variables of interest were expressed as percentages and presented in tables.

**Ethical clearance**: Ethical clearance was gotten from the Institutions ethical board and the ethical principles of non-maleficence, beneficence, confidentiality and respect of persons were applied throughout the duration of the study. The patients were well counseled on the purpose of the study and they all gave consent. Those who withheld consent were excluded from the study.

## Results

Severe intrauterine adhesion accounted for 19 (25.0%) out of 76 cases of intrauterine adhesions managed during the period. This constituted 11.9% of 160 infertile women who had diagnostic hysteroscopies in our units over the study period. The mean age was 36.3+/-4.3 years and all the women belong to the parity group of 0-1. The mean duration of symptom was 4.2+/-3.2.years (Table 1). Amenorrhea in association with infertility (68.4%) was the main presenting complaint while the major risk factors were complicated caesarean section (42.1%) and abdominal myomectomy (26.3%). On hysteroscopy, the adhesions were mainly dense (52.6%) and multiple (94.7%) with complete involvement of the uterine cavity in all the cases. Obliterative lesions were seen in 63.2% of the women. There were previous attempts at correction in 31.6% of the women which comprised of blind adhesiolysis with uterine sound in all cases (Table 2). Table 3 shows the characteristics of severe intrauterine adhesions as seen on hysteroscopy. The adhesions were mainly dense (52.6%), multiple (94.7%) and obliterative (63.2%). There was complete (100.0%) involvement of the uterine cavity in all the cases while the cervical os was involved in 68.4% of cases. Complications were encountered in 2 (10.5%) of the women.

**Table 1:** Distribution by sociodemographic characteristics of the infertile Nigerian women with severe IUA

Age range	Freq.	Percent
25-29	2	10.53
30-34	4	21.05
35-39	11	57.89
40 and above	2	10.53
**Occupation**		.
Public servant	8	42.11
Health worker	7	36.84
Trading	2	10.53
Student	2	10.53
**Religion**	Freq.	Percent
Catholic	12	63.16
Anglican	7	36.84
**Husband’s occupation**		
Trader	11	57.89
Public servant	2	10.53
Artisan	4	21.05
Health worker	2	10.53
**Highest educational status**		
Primary	2	10.53
Secondary	6	31.58
Tertiary	11	57.89

**Table 2:** Distribution by the clinical presentation of the infertile Nigerian women with severe IUA

Mode of presentation	Freq.	Percent
Amenorrhea and infertility	13	68.4
Hypomenorrhea	3	15.8
Amenorrhea	3	15.8
**Risk factors/aetiology**		
C/S	8	42.1
D & C for evacuation	2	10.5
Myomectomy	5	26.3
PID	2	10.5
MVA	2	10.5
**Duration of symptoms**		
2 years and below	11	57.9
More than 2 years	8	42.1
Pelvic surgery		
Yes	15	78.9
No	4	21.1
**Dysmenorrhea**		
yes	2	10.5
No	17	89.5
**Any cyclical abdominal pain**		
Yes	6	31.6
No	13	68.4
**Pelvic surgery**		
Yes	15	78.9
No	4	21.1
**Previous attempt at correction**		
No	13	68.4
Yes	6	31.6
**Complications**		
Cervical Lacerations	2	10.5
None	17	89.5

**Table 3:** Distribution by hysteroscopic characteristics of severe intrauterine adhesions seen among infertile Nigerian women

Nature of the adhesion bands	Freq.	Percent
Dense	10	52.6
Dense & filmy	9	47.4
**Number of adhesions**		
Multiple	18	94.7
Single	1	5.3
**Distribution of the adhesions**		
Cavity & Periostial	6	31.6
Cavity, Internal os & Periostial	13	68.4
**Configuration of the adhesion bands**		
Obliterative	12	63.2
Obstructive	3	15.8
Combination	4	21.1
**Extent of cavity involved**		
Complete	19	100.0
**Obliteration of the cervical os**		
Yes	13	68.4
No	6	31.6
**Use of Resectoscope**		
Yes	15	78.9
No	4	21.1

## Discussion

The management of severe intrauterine adhesions (IUAs) is challenging and associated with poor reproductive outcomes and a high recurrence rate. In our study, severe intrauterine adhesion accounted for 19(25.0%) out of 76 cases of IUAs and 11.9% of all infertility patients who had diagnostic hysteroscopies in our units over the study period. This significant number of severe may not represent the true prevalence in our environment as our unit is a referral unit for gynaecological endoscopy but it has implications for management outcomes for the patient as severe IUAs are known to be associated with poor reproductive outcomes following surgery in terms of menstrual return, pregnancy rate and live birth rates [12-13]. It therefore means that there is need to acquire the necessary skills for the surgical treatment of these cases as the other options of fertility management in these women which may include gestational surrogacy and adoption in our environment. The major aetiological events/risk factors for severe IUAs as found in our work were caesarean section and myomectomy. In the previous reports from Nigeria, dilatation and curettage was the main risk factor identified [1-4]. But the women were not classified in terms of severity of the symptoms. It is known that intrauterine adhesions following caesarean section occur within the context of caesarean section complicated either by sepsis or postpartum haemorrhage warranting excessive uterine curettage. Therefore, maintaining asepsis, the use of prophylactic antibiotics as well as good surgical skills to manage postpartum haemorrhage will reduce the incidence of IUA following caesarean section. Abdominal myomectomy is another major risk factor from our work. Although many authors have reported on the relationship between peritoneal adhesions and myomectomy [27], only a few have evaluated the role of myomectomy in causing IUAs [28-30]. The incidence of IUAs following abdominal myomectomy ranges from 21.5% to 50% [29-31]. Damage to the endometrium is considered the aetiopathogenetic pathway to IUAs following open myomectomy. Gambadauro et al in 2012, [28] reported a significantly higher occurrence of IUA when the endometrium was breeched. However, in contrast, IUAs have also been found following myomectomies in which the endometrium was not breeched [31].

In these cases, tissue ischemia and vascular compromise arising from extensive tissue dissection are thought to be responsible for IUAs. This significant association of myomectomy with IUAs underscores the need to adopt measures to limit the occurrence of intrauterine adhesions post myomectomy. This is especially important as myomectomy is mostly done for low parity women, most of whom still desires further pregnancy. The main clinical presentation of women with IUA found in our work is amenorrhea. This is in line with the hysteroscopy finding of complete cavity involvement in almost all the cases. Complete occlusion of the internal cervical os can also lead to amenorrhea and is particularly of concern as it makes HSG evaluation of the cavity impossible. Secondary dysmenorrhea and cyclical abdominal pain were found in a small number of the women. In an earlier report by Takai et al [3] in Nigeria, most of the patients presented with hypomenorrhea and infertility. However, the patients in their work were not graded as diagnosis was made by hysterosalpingogram. The adhesion bands were mainly dense adhesions which were mainly multiple involving the whole of the cavity. The remaining was a mixture of filmy and dense adhesions. Dense adhesion bands are very tough fibrotic bands that are difficult to resect with the scissor thereby increasing the use of the electrosurgical systems with the attendant increased risk of endometrial damage and recurrence. It is not surprising then, that monopolar resectoscope was used for resection in majority (78.9%) of the cases. Also the adhesion bands were mostly complete obliterative lesions. Typically, these lesions present with amenorrhea which may be associated with cyclical abdominal pain in a few cases where there are still small endometrial tissues left. Cyclical abdominal pain found in 31.6% of the women would likely be majorly on account of the occlusive lesions and to a small extent, the obliterative lesions. The hallmark of presentation of the occlusive lesion is reduced/absent menstrual flow in association with cyclical abdominal pain. Our complication rate was minimal and constitute mainly of cervical lacerations. Complications are increased during operative hysteroscopies for severe intrauterine adhesions and may be in the form of cervical lacerations, uterine perforations and haemorrhage as well as anaesthetic and fluid overload complications. The use of ultrasonography and laparoscopy as guides during the procedure may have been responsible for the reduced rates of complications in our work.

**Limitation**: Our main limitation was the fact that this was a hospital based study and therefore the findings may not be generalized. However, the study gave a very useful insight into the problem of severe intrauterine adhesions in our area as our units are referral units for infertility management.

## Conclusion

The prevalence of severe IUA is high among women with IUAs and presents mainly with amenorrhea and infertility. The main risk factors for severe IUA are complicated caesarean section and myomectomy. There is need to build capacity for hysteroscopic management of intrauterine adhesions in Nigeria to improve outcome.

### What is known about this topic

- The prevalence, risk factors, mode of presentation and reproductive outcomes following treatment for severe intrauterine adhesions among the developed countries.

### What this study adds

- Severe intrauterine adhesions accounted for 25.0% of all cases of intrauterine adhesions managed during the period. Amenorrhea in association with infertility (68.4%) was the main presenting complaint while secondary dysmenorrhea and cyclical abdominal pain were other features. The main aetiological events were complicated caesarean section (42.1%) and abdominal myomectomy (26.3%).

## Competing interests

The authors declare no competing interests.
